# The Influence of Empathy and Morality of Violent Video Game Characters on Gamers’ Aggression

**DOI:** 10.3389/fpsyg.2017.01863

**Published:** 2017-11-14

**Authors:** Xuemei Gao, Lei Weng, Yuhong Zhou, Hongling Yu

**Affiliations:** ^1^Faculty of Psychology, Southwest University, Chongqing, China; ^2^Key Laboratory of Cognition and Personality, Ministry of Education, Southwest University, Chongqing, China

**Keywords:** violent video games, justice, aggression, empathy, morality, game character

## Abstract

According to the General Aggression Model, situational factors (such as the game characters) and personal factors both affect a gamer’s acquisition of aggressive behavior. Previous studies have found not only that the surface features of game characters, such as appearance and clothing, but also that their inherent characteristics, such as morality and identity, can influence a gamer’s attitude and behavior. Research has also shown that empathy, as a personal factor, can protect gamers from the impact of media violence. However, past research has focused primarily on single factors affecting the player rather than more comprehensive investigations. This study investigates the influence of the game character’s moral features and levels of empathy on the gamer’s aggression. The participants were 120 Chinese university students (61 females and 59 males) with ages ranging from 17 to 27 years. Participants first completed a series of questionnaires: a user experience questionnaire, a video game questionnaire, the Buss-Perry Aggression Questionnaire, and a modified version of the Interpersonal Reactivity Index. All participants then had 5 min of practice playing a violent video game. They were then divided into three groups: a high empathy group, a low empathy group, and a no empathy group. After the practice, participants in the high and low empathy groups read empathy materials relating to the game characters; participants in the no empathy group began formal gameplay. All participants played the game for 20 min. Finally, participants were required to complete the Scale of Hostility Status questionnaire, the Implicit Aggression Test, and the Competitive Reaction Time Test. The results show that empathy and the morality of game characters both influence aggression, but empathy affected aggression differently in the participants playing justified roles (i.e., killing others for a moral reason in the game) compared to those playing unjustified roles (i.e., killing others for immoral reasons in the game). In the high empathy condition, the implicit aggression of justified players was significantly higher than those playing unjustified roles. However, high empathy does not always play a protective role, and its effect is restricted by the features of the game characters.

## Introduction

The game character, which defines the role that an individual plays in a video game, is the primary means for a player’s interaction with the virtual environment (Shapiro et al., 2006). Game characters are indispensable to the game and are able to affect a player’s behavior. Players can be influenced by the physical characteristics of the game character and also by the character’s implicit attributes, both of which can affect the level of a player’s personal involvement and their identity within the game. With the rapid development of the video game industry, the picture quality of the game scene is increasingly more delicate while game characters are becoming ever more realistic. As a result, players can better acquaint themselves with the game characters they play and the game as a whole.

Studies have found that the features of the game characters in violent video games (such as appearance, race, costume, and moral attributes) have an effect on the players. The more attractive the appearance of the game characters, the more confident the players are in the social context; as a result, the players have closer relationships with other players in the game, which shapes stronger interactions (Yee and Bailenson, 2007). The players in black garments display a stronger tendency toward aggression in virtual tasks and weaken group cohesion (Johnson and Downing, 1979; Meier et al., 2004; Peña et al., 2009). Individuals playing Black characters tend to have more negative assessments and exhibit more aggressive behavior after playing a game (Yang et al., 2014). Research has also shown that characters from different countries can have an effect on individuals. A study by Alhabash and Wise (2012), in which the research subjects were required to play characters acting against their country in an educational electronic game, found that this could promote attitude changes to a certain degree.

Many aspects of the game characters are influential: not only surface features like appearance and clothing, but also significant characteristics like morality and identity. Morality is the core attribute of game characters. Many researchers have explored this issue by giving game characters different types of in-game instructions, such as invading other countries for justifiable or unjustifiable reasons (Tamborini et al., 2001; Schneider et al., 2004). The results showed that, when a player acted as a “good” game character in a violent video game (such as Righteous Army or Counter-Strike), the moral panic and negative emotional experience generated by playing games was less (Hartmann and Vorderer, 2009; Hartmann et al., 2010; Weaver and Lewis, 2012; Shi et al., 2013). Research also showed that empathy lowered aggression in the justified-role condition, but enhanced aggression in the unjustified-role condition (Happ et al., 2013). Additionally, players in the unjustified-role condition had more worries, pains, and other negative emotional experiences (Happ et al., 2013). Studies have also shown that both moral anxiety and negative emotions decrease when individuals play in the justified roles in, for instance, Ally of Justice and Counter-Strike (Schneider et al., 2004).

From the trait empathy perspective, empathy is a crucial mediation variable between violent video game exposure and aggressive behavior (Calvert et al., 2006; Zhen et al., 2011). Research has shown that individuals with high trait empathy are opposed to unjustified violent behavior (Hartmann et al., 2010). Therefore, some researchers have suggested that empathy can be treated as a protective factor that adjusts the influence of media violence on individuals and can weaken the negative effects of video games (Miller and Eisenberg, 1988; Zhen et al., 2011).

According to the General Aggression Model (GAM) (Anderson and Bushman, 2002), personal factors and situational factors affect not only the internal status of individuals (such as cognition, affection, and physiological activation), but also their decision-making and subsequent behaviors. Studies indicate that the relevant personal factors making players more offensive include personality traits (Markey and Markey, 2010), empathy (Funk et al., 2004). Situational factors include game content, such as violent or prosocial tendencies (Anderson et al., 2004; Adachi and Willoughby, 2011); platform (factors such as 2D or 3D, gamepad, and motion controller); settings (e.g., game scene and game characters); and play mode, such as cooperative or competitive (John et al., 2014). Gentile (2011) argues that these factors do not function separately but instead interact.

However, past research has focused primarily on a single factor affecting the player and failed to conduct a more comprehensive investigation, such as the interaction of two factors. Further, there is debate about the relationship between violent video games and empathy and aggression (Gao et al., 2017; Hilgard et al., 2017; Kepes et al., 2017; Szycik et al., 2017). To remedy this deficiency, the present study investigates the influence of the moral attributes of game characters and that of empathy in the players, as well as their interactive effect. The research investigates the effect of different levels of empathy on aggression specifically by inducing empathy during gameplay. On the basis of GAM and previous findings, the study proposes the following hypotheses:

Hypothesis 1: Following gameplay, the aggression of unjustified-role players will be higher than that of justified-role players.
Hypothesis 2: In justified-role players, the aggression of individuals with high or low levels of empathy will be lower than the aggression of those without empathy. The opposite will be seen in unjustified-role players.
Hypothesis 3: The aggression of individuals with high empathy will be significantly lower than the aggression of those with a low level of empathy.

## Materials and Methods

### Participants

Hundred and twenty college students were recruited online to volunteer in this experiment. The ages of the participants ranged from 17 to 27 years (*M* = 21.01, *SD* = 1.65). The participants included 61 females (*M* = 21.17, *SD* = 1.63) and 59 males (*M* = 20.66, *SD* = 1.65). In the formal experiment, participants were randomly assigned to one of six conditions in a 2 × 3 between-subjects design (character: Justice vs. Injustice × empathy: high vs. low vs. no).

Advertisements for recruiting participants were posted on Internet community sites and stated the remuneration for the research, which would be paid following participation. Individuals interested in the study could send their personal information and contact details to the researcher. The researcher selected 120 persons from the applicants to join the study.

### Video Game (Mortal Kombat3)

Mortal Kombat 3 (MK3) is a fighting game developed by Midway Games. As in previous games in the series, it has a cast of characters for players to choose from; they are then guided through a series of battles against other opponents. Background information about these main characters is readily available, which was helpful in compiling empathy materials.

### Empathy Materials

We selected two game characters, Nightwolf and Rain, to represent the justified and unjustified characters respectively, and to serve as the leading roles in empathy materials. In high empathy materials, Nightwolf was raised in an Indian tribe filled with love and harmony, and then joined a justified alliance to fight against invaders. Conversely, Rain grew up in a training camp full of violence and killing, and later followed an unjustified alliance in invading other countries. In the low empathy materials, only the appearance, skills, and allegiances of the characters were described.

### Measurements

#### Interpersonal Reactivity Index-C (IRI-C)

This scale is a revised form of the scale proposed by Davis (1980). It includes 22 items divided into four dimensions; namely, PT (perspective taking), FS (fancy sympathy), EC (empathic concern), and PD (perspective distress). The scale uses a five-point scoring system from 0 to 4. The higher the score, the stronger the empathic ability of the individuals. The Cronbach’s α of the IRI-C was 0.81 (CI 90 = 0.77 to 0.84).

#### Buss-Perry Aggression Questionnaire (BPAQ)

This scale is a revised version of the scale proposed by Buss and Perry (1992) to measure an individual’s level of aggression. It includes 29 items in total divided into four dimensions: physical aggression (PA), verbal aggression (VA), anger (A) and hostility (H). A five-point scoring system was used in this questionnaire, and the higher the score, the stronger the aggression level of the individual. Cronbach’s α was 0.83 (CI 90 = 0.79 to 0.89) at pretest, and 0.89 (CI 90 = 0.85 to 0.91) at post-test.

#### Scale of Hostility Status (SHS)

Scale of Hostility Status is a revised version of a questionnaire compiled by Anderson et al. (1995) and is used to measure the empathy level of an individual’s characteristics, with improved reliability and validity (Cronbach’s α = 0.84). The Chinese scale includes a total of 29 questions. The scale adopts a five-point scoring system from 1 to 5. The higher the score, the stronger the individual’s hostility.

#### Aggressiveness Implicit Association Test (IAT)

Aggressiveness Implicit Association Test is a simple classification task that measures implicit aggression. It is based on the reaction time of individuals and reflects automatic attitudes, stereotypes, self-esteem, and trait components of an individual’s self-concept (Greenwald and Banaji, 1995). There are two steps to this test: implicit aggression concept and implicit aggression evaluation. Both tasks contain seven blocks, with block 4 and block 7 being formal phases. Additionally, block 4 is the compatible task, while block 7 is the incompatible task. Data were analyzed using the algorithm proposed by Greenwald (Greenwald et al., 2003): the D measure. The higher the d-score, the stronger the implicit aggression.

#### Competitive Reaction Time Test (CRTT)

Used over 25 trials, CRTT measures an individual’s aggression. Before each trial, subjects are required to set noise punishing levels for pretend rivals, including intensity and duration. The 10 grades range from 0 to 9. At the start of each trial, a pure tone is presented and the subject should compete to press the K on the keyboard as fast as possible. Competing results are then presented to the subject. If the subject was slower than his rival, he receives punishment set by that rival. This was considered a failure. Conversely, if the subject succeeded, the trial proceeded. The average score of noise strength and noise duration set by the subjects in each trial is calculated separately, and the mean score of the two averages indicates the individual’s aggression.

### Research Procedures

Following the selection of the participants, the subjects signed an informed consent form and provided socio-demographic information. They were then asked to complete a user experience questionnaire, a video game questionnaire, BPAQ, and IRI-C. Subjects were then randomly allocated to act as the justified (Nightwolf) and unjustified (Rain) game characters, and evaluated their familiarity with the game and the characters using a seven-point grading system (1 = very unfamiliar; 7 = very familiar). Subjects were allowed to practice the game for about 5 min to establish a good command of the basic game operations. In the game, the basic task was to attack people on the street. After the practice session, the no-empathy group began formal gameplay. Subjects in the high and low empathy groups were required to read the materials relating to the game character. Afterward, these subjects were required to play the game for 20 min. Once the game was over, they completed the SHS, IAT, and CRTT questionnaires.

## Results

### Statistical Analysis of Control Variables

An analysis of variance (ANOVA) of violent video game exposure in six conditions was conducted and the results were found to be non-significant [*F*(5,113) = 0.97; *p* > 0.05; *d* = 0.34], suggesting no significant difference between exposure to high violent content and exposure to low violent content. The result of the IRI-C ANOVA was also non-significant [*F*(5,113) = 0.40; *p* > 0.05; *d* = 0.15], indicating no significant difference among groups in trait empathy. The difference of trait aggression between groups was also non-significant [*F*(5,112) = 1.09; *p* > 0.05; *d* = 0.38], suggesting no differences in aggression traits across individuals. However, the difference of familiarity with the game was significant [*F*(5,113) = 2.74; *p* < 0.05; *d* = 0.81], as was the difference of familiarity with the game characters [*F*(5,113) = 2.55; *p* < 0.05; *d* = 0.77]. To avoid confounding variables, these two factors were taken into subsequent analysis as being covariant.

### Aggressive Cognition

Taking the empathy level and moral attributes of the game characters as independent variables, the familiarity with the game and game characters as covariant, and the implicit aggression and implicit aggressive evaluation as dependent variables, a multivariate analysis of variance (MANOVA) showed non-significant effects of both moral attributes [*F*(1,110) = 0.32; *p* > 0.05; *d* = 0.09] and empathy [*F*(2,110) = 0.29; *p* > 0.05; *d* = 0.04] on implicit aggression. However, the interaction between moral attributes and empathy was significant [*F*(2,110) = 3.55; *p* < 0.05; *d* = 0.65].

Furthermore, simple effect analysis showed that the difference of implicit aggression between justified-role and unjustified-role players was significant [*F*(2,110) = 6.37; *p* < 0.05; *d* = 0.71], while the implicit aggression in justified-role players (-0.01 ± 0.07) was higher than in unjustified-role players (-0.26 ± 0.07). Additionally, the difference of implicit aggression between high empathy and low empathy conditions approached significance in the justified- role play condition (*p* = 0.05). The implicit aggression of high empathy individuals (-0.01 ± 0.07) was higher than that of low empathy individuals (-0.21 ± 0.07). The results are shown in **Table 1**.

**Table 1:** The mean scores (M) and standard deviation (SD) of each variable.

Empathy	AC (IAT1)		AC (IAT2)		AE (SHS)		AB (NS)		AB (ND)	
	J	I	J	I	J	I	J	I	J	I
High	–0.01 (0.07)	–0.26 (0.07)	–0.48 (0.08)	–0.58 (0.07)	74.60 (3.33)	74.52 (3.43)	4.59 (0.49)	3.96 (0.46)	3.16 (0.46)	3.12 (0.45)
	*F =* 41.67 *p =* 0.000^∗∗∗^ *d =* 0.82		*F =* 5.392 *p =* 0.049^∗^ *d =* 0.55		*F =* 0.011 *p =* 0.918 *d =* 0.08		*F =* 6.421 *p =* 0.035^∗^ *d =* 0.63		*F =* 0.038 *p =* 0.851 *d =* 0.05	
Low	–0.21 (0.07)	–0.15 (0.07)	–0.71 (0.07)	–0.63 (0.07)	80.97 (3.51)	79.26 (3.33)	4.56 (0.48)	3.61 (0.49)	3.28 (0.46)	3.28 (0.51)
	*F =* 4.168 *p =* 0.075 *d =* 0.34		*F =* 9.205 *p =* 0.016^∗^ *d =* 0.59		*F =* 1.018 *p =* 0.342 *d =* 0.20		*F =* 22.403 *p =* 0.001^∗∗∗^ *d =* 0.49		*F =* 0.006 *p =* 0.940 *d =* 0.13	
No	–0.18 (0.07)	–0.09 (0.08)	–0.54 (0.08)	–0.44 (0.07)	83.35 (3.36)	83.68 (3.37)	4.81 (0.48)	4.15 (0.45)	3.53 (0.45)	3.35 (0.46)
	*F =* 6.769 *p =* 0.032^∗^ *d =* 0.67		*F =* 7.139 *p =* 0.028^∗^ *d =* 0.71		*F =* 0.013 *p* = 0.911 *d =* 0.20		*F =* 9.618 *p =* 0.015^∗^ *d =* 0.74		*F =* 0.809 *p =* 0.395 *d =* 0.16	

*AC (IAT1), Aggressive Cognition (Implicit Aggression); AC (IAT2), Aggressive Cognition (Implicit Aggression Evaluation); AE (SHS), Aggressive Emotion (Scale of Hostility Status); AB (NS), Aggressive Behaviors (Noise Strength); AB (ND), Aggressive Behaviors (Noise Duration); J, Justice; I, Injustice. ^∗^*p* < 0.05, ^∗∗^*p* < 0.01, ^∗∗∗^*p* < 0.001.*

**FIGURE 1:**
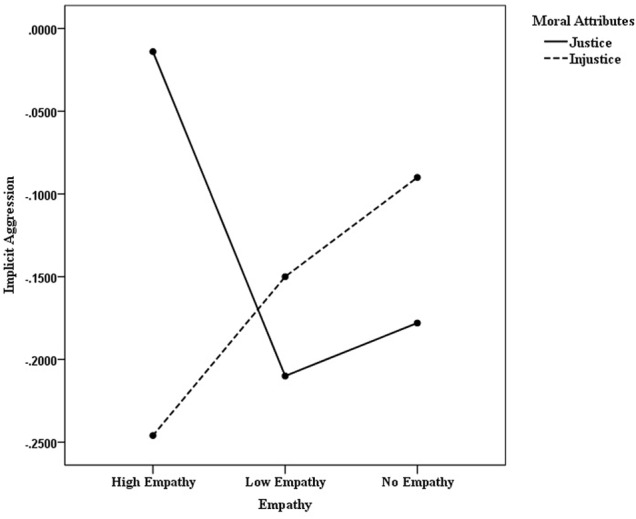
The interaction between empathy and moral attributes of game characters on implicit aggression.

The effect of moral attributes on implicit aggression evaluation was non-significant [*F*(1,110) = 0.17; *p* > 0.05; *d* = 0.07], whereas the effect of empathy was significant [*F*(2,110) = 3.32; *p* < 0.05; *d* = 0.62]. The Bonferroni Test showed that the difference between the effects of high and low empathy conditions on implicit aggression evaluation approached has significant marginal difference (*p* = 0.059), with the former (-0.53 ± 0.05) higher than the latter (-0.67 ± 0.05), the difference between the low and no empathy conditions was also significant (*p* = 0.017). Lastly, the interaction between moral attributes and empathy was found to be non-significant [*F*(2,110) = 1.01; *p* > 0.05; *d* = 0.22], as shown in **Figure 1**.

### Aggressive Emotion

Multivariate analysis of variance was conducted with the empathy level and moral attributes of the game characters as the independent variable, the familiarity with the game and game characters as covariant, and the SHS scores as the dependent variable. Results showed that both moral attributes [*F*(1,110) = 0.02; *p* > 0.05; *d* = 0.05] and empathy [*F*(2,110) = 3.28; *p* < 0.05; *d* = 0.61] had no significant effect on SHS scores. The Bonferroni Test showed a significant difference between the high and low empathy conditions on SHS (*p* = 0.013). Furthermore, hostile emotion in the high empathy condition (74.56 ± 2.42) was lower than in the no empathy condition (83.42 ± 2.40). Lastly, there was no significant interaction between moral attributes and empathy [*F*(2,110) = 0.06; *p* > 0.05; *d* = 0.06].

### Aggressive Behaviors

Another MANOVA was conducted with the empathy level and moral attributes of the game characters as the independent variable, the familiarity with the game and game characters as covariant, and the strength of the noise and its duration (in CRTT) as the dependent variable. On the one hand, the effect of moral attributes on the strength of the noise set by individuals was significant [*F*(1,110) = 3.85; *p* < 0.05; *d* = 0.49], with those who played justified roles (4.65 ± 0.28) setting higher levels than those who played unjustified roles (3.91 ± 0.26). However, there was no significant effect of empathy [*F*(2,110) = 0.34; *p* > 0.05; *d* = 0.10], and no significant interaction found between moral attributes and empathy [*F*(2,110) = 0.07; *p* > 0.05; *d* = 0.06]. As regards duration, there was no significant effect of moral attributes [*F*(1,110) = 0.04; *p* > 0.05; *d* = 0.05] or empathy [*F*(2,110) = 0.20; *p* > 0.05; *d* = 0.08] on the duration of the noise set by individuals. Finally, the interaction between moral attributes and empathy was also non-significant in this case [*F*(2,110) = 0.02; *p* > 0.05; *d* = 0.05].

## Discussion

This research investigated the effects of the moral attributes of game characters and empathy levels on player aggression. Results showed that the moral feature itself can influence player aggression: more aggressive behaviors were emerging among players in justified roles, which was contrary to our first hypothesis and inconsistent with previous findings of how game characters affect individuals (Meier et al., 2004; Peña et al., 2009; Happ et al., 2013). Previous studies have indicated that behaving badly in a game increases aggression and reduces helping behavior and group cohesion. This disagreement in the findings may be due to two reasons. Firstly, the game characters used in previous studies were all famous. Calvert et al. (2004) found that there was a stronger impact with well-known game characters, which players tended to identify with and imitate. Happ et al. (2013) used Superman in his experiment, and Peña et al. (2009) used black-cloaked avatars. It is easier for individuals to predict a game character’s behavior and behave similarly when playing a famous role (Bargh et al., 1996). However, when playing lesser-known roles, more information is required for players to make decisions and this affects the results (Bargh et al., 1996). Secondly, the in-game behaviors representing justice are considered rational to game players.

Relevant research showed that rejection of violent behavior was reduced if the violent behavior was justified (Funk et al., 2004). Additionally, negative emotions like anxiety following the consequences of violent behavior could be less (Hartmann and Vorderer, 2009; Weaver and Lewis, 2012). For the majority, “justice” implies correctness, and it is deemed acceptable if heroic characters treat others violently. This strengthens identification with the aggressive behaviors of justified characters and leads to similar behavioral patterns post-game.

The main result of this study is that empathy remitted aggression to a certain extent. High and low empathy conditions had different effects: the reduction of aggression in the low empathy condition was not as evident as that in the high empathy condition for hostile emotions. Furthermore, in implicit aggression evaluation, aggressive cognition in the low empathy condition clearly dropped and was higher in the high empathy condition than in the low empathy condition. This indicates that the difference in empathy could be an important factor affecting the function of empathy within the game.

The interaction between empathy and moral attributes showed that the implicit aggression of players fulfilling just roles was higher than that of those playing unjust roles. Furthermore, the effect of empathy differed across different moral attributes of the game characters. Although the effect was not very obvious, empathy could have eased player aggression when they played unjust roles. Conversely, empathy enhanced player aggression playing just roles, the effect of which was more obvious in the high empathy condition. This is consistent with the findings made by Happ et al. (2013, 2014) and shows that the effect of empathy in video games can be affected by game character features. A possible reason for this could be that the empathy materials, describing the features of the game characters, provided a basis for justifying the character’s aggressive behavior, thereby making players more inclined to judge the behavior of just game characters as being reasonable, and enhancing their aggression. Previous studies concerning priming materials have demonstrated this to be the case (Schneider et al., 2004; Lin, 2013a,b).

In addition, the difference in aggression between high and low empathy conditions resulted from content discrepancy and manipulation of the empathy materials. In the low empathy materials, there was less description about role characteristics and more objective illustration, which weakened the degree of empathy and rationalization of behavior. Therefore, the impact of low empathy materials on aggression was not as apparent as with the high empathy materials.

This paper’s findings show that the influence of empathy on the aggression of the game players was moderated by the game characters. Empathy strengthened player attitudes (Happ et al., 2013) and amplified the difference between “justice” and “injustice,” aggression changing correspondingly. Furthermore, empathy and moral attributes can affect player aggression, while the effect of empathy in specific situations is restrained by other factors. Our findings also suggest that investigating the influence of single factors provides a fragmentary perspective, while taking multiple factors into consideration is more systematic and leads to a better understanding of the underlying mechanisms. Lastly, our research helps us to understand how to eliminate the negative effects of video games and how to aggrandize their positive influence and applications in other fields.

### Strengths and Limitations

There are several limitations to the present study. First, the adoption of online recruitment in the study may lead to a self-selection bias. It means researchers have no control over the process of selecting participants; rather, participants selected themselves for the study, which undermines the external validity of the study and the interpretation of the findings. Secondly, we found the positive effect of empathy was not very significant. A random selection of participants were interviewed after gameplay, and some of them reported that the content of the empathy materials was not very realistic, which may constrain the effect of empathy (Decety et al., 2010). The findings of this experiment therefore require further verification.

Despite these limitations, we believe that our study sheds more light on the question of how empathy and the morality of violent video game characters influence gamers’ aggression. The moral characteristics of the game characters seem to particularly affect gamers’ aggression, with more aggressive behaviors emerging in the justice condition than in the injustice condition. Meanwhile, empathy amplified the effect, boosting the negative effect in the justice condition, and moderating the negative effect in the injustice condition.

Further studies should investigate the underlying mechanism of inducing empathy. In the present study, it is not possible to know whether the effect of empathy was due to its changing the level of identification with the game character, due to its affecting only moral concerns, or due to a combination of the two. Previous researches have also suggested that identification with a violent video game character (Konijn et al., 2007) and moral disengagement in a violent video game (Hartmann et al., 2010) are important aspects in explaining the negative effects of violent video games. A further consideration is that our study investigated only the short-term effects of empathy and the morality of violent video game characters; future research should explore whether there are long-term effects.

## Ethics Statement

All procedures involving human participants in this study were conducted in accordance with the ethical standards of the institutional and/or national research committee and with the 1964 Declaration of Helsinki and its later amendments or comparable ethical standards. The Institutional Review Board at Southwest University in Chongqing, China approved all procedures. The study protocol was approved by the Ethics Committee of Southwest University. Written informed consent was obtained from all individuals participating in the study after a detailed explanation of the study protocol. The Institutional Review Board at Southwest University approved this consent procedure.

## Author Contributions

XG and LW conceived and designed the experiments. LW performed the experiments. LW, XG, and YZ analyzed the data. XG, LW, YZ, and HY wrote the paper.

## Conflict of Interest Statement

The authors declare that the researchwas conducted in the absence of any commercial or financial relationships that could be construed as a potential conflict of interest.
